# The Paradox of Minor Pain: How Physical Pain Shapes Loneliness in Small Networks with Gender Differences

**DOI:** 10.1093/geroni/igaf122.506

**Published:** 2025-12-31

**Authors:** Haosen Sun

**Affiliations:** University of Nevada, Reno, Reno, Nevada, United States

## Abstract

Loneliness in older age is widely understood as a subjective experience stemming from the gap between expected and actual levels of social connectedness. However, how this psychological discomfort intersects with physical pain in older adults—and whether these patterns differ by gender—remains unclear. Pooling data from Wave 4 (2011) and Wave 6 (2015) of the Survey of Health, Aging, and Retirement in Europe (SHARE), which provides detailed information on social network composition and self-reported pain, we conducted linear regression analysis with a longitudinal setup to examine whether individuals experiencing pain are more likely to report higher levels of loneliness compared to those with similar network sizes but without pain, with particular focus on those in smaller networks. The analysis controlled for baseline loneliness, baseline pain, network changes, social activities, personality traits, other health factors, and key sociodemographic variables. We also explored whether this intersection varies between males and females. Among individuals with small networks (fewer than two core confidants), those reporting minor pain experienced even lower levels of loneliness than their counterparts without pain, suggesting that minor discomfort may reduce expectations or justify a smaller network. In contrast, individuals experiencing moderate or severe pain reported significantly higher levels of loneliness with smaller networks, highlighting an increased need for support when pain becomes more intense. This pattern was particularly pronounced among males but not females, suggesting potential gendered coping mechanisms. Men may place greater expectations on external support and experience greater emotional vulnerability when such support is lacking when needed.

